# Sarcomatoid hepatocellular carcinoma mimicking hepatic abscess: A case report

**DOI:** 10.1097/MD.0000000000022489

**Published:** 2020-09-25

**Authors:** Zheng Yang, Kun Lv, Yanan Zhao, Minqiang Pan, Chao Zhang, Shumei Wei

**Affiliations:** a Department of Anesthesiology; b Department of Ultrasound; c Department of Pathology, the Second Affiliated Hospital of Zhejiang University School of Medicine, Hangzhou, Zhejiang, China.

**Keywords:** cholangiocarcinoma, hepatocellular carcinoma, liver abscess, sarcomatoid hepatocellular carcinoma, ultrasound

## Abstract

**Rationale:**

Primary sarcomatoid hepatocellular carcinoma (SHC) is a rare subtype of morphologic hepatocellular carcinoma reported on less than 1% of surgical pathology specimens. Herein, we report a rare case of SHC. The case in question was initially misdiagnosed as a liver abscess due to the clinical and radiological similarity between these 2 pathologies. Ultrasound(US)- and contrast-enhanced ultrasound (CEUS)- guided biopsies are helpful in making an accurate diagnosis under the appropriate biopsy area and angle of puncture.

**Patient concerns:**

A 56-year old male presented to our hospital with a 2-month history of dull, upper abdominal pain without radiation.

**Diagnoses:**

Upon initial investigation with computed tomography, a cystic mass was found in the hepatic V segment and an infectious etiology was presumed. Further diagnostic examination with CEUS and magnetic resonance imaging suggested a hepatic abscess. However, a diagnosis of atypical intrahepatic cholangiocarcinoma was not excluded. The patient received the standard antibiotic treatment without alleviation of his symptoms. Through 3 diagnostic US-and CEUS-guided biopsies over a 3-month period, the pathological diagnosis of SHC was finally confirmed.

**Interventions:**

The patient was diagnosed by 3 diagnostic US-and CEUS-guided biopsies, the pathological diagnosis of SHC was finally confirmed.

**Outcomes:**

Due to the delay in diagnosis, the patient was not a candidate for surgical resection, and showed dissemination of the lesion to the portal vein. Therefore, treatment with chemotherapy was initiated. After 4 courses of this regimen, tumor progression was found on enhanced magnetic resonance imaging. Therefore, the patient received immunotherapy and targeted therapy with limited response. The patient passed away 3 months later due to tumor progression.

**Lessons:**

A hepatic abscess should be considered as a malignant lesion when clinical symptoms do not resolve upon standard treatment. US- and CEUS- guided biopsies are helpful in making an accurate diagnosis under the appropriate biopsy area and angle of puncture.

## 1 Introduction

Primary Sarcomatoid hepatocellular carcinoma (SHC) is a rare subtype of morphologic hepatocellular carcinoma (HCC) and is reported on less than 1% of surgical pathology specimens.^[1]^ As such, only a small number of cases have been reported in the literature.^[2,3]^ SHC is recognized as a clinically aggressive carcinoma with a poor prognosis and low 5-year survival rate. Primary presentation of malignancy often occurs as a liver abscess and is mainly described in patients with metastatic colorectal cancers, intrahepatic cholangiocarcinoma (ICC) or neuroendocrine tumors.^[4–6]^ The accurate diagnosis of SHC is very difficult and without timely and reasonable surgical management, tumors could progress rapidly and miss the chance of surgery. To improve the understanding and guide diagnosis for SHC, this case report retrospectively analyzed the misdiagnosis factors of the rare case of SHC.

## 2 Case report

In February 2019, A 56-year old male presented to our hospital with a 2-month history of dull upper abdominal pain without abdominal distention, nausea or vomiting, and an absence of an inciting event prior to onset. Work-up with computed tomography (CT) in local hospital suggested an infectious hepatic lesion in the hepatic V segment. The pain was not alleviated following antibiotic therapy and the patient then presented to our hospital for further treatment. The patient was admitted to the inpatient ward with a space-occupying lesion of the liver. The patient has a medical history of Hepatitis B for 1 year, treated with Lamivudine, 1 tablet a day. The patient does not have a family history of SHC or HCC. Upon physical examination, abdominal tenderness was noted. The exam was negative for rebound tenderness, muscular tension, nausea, vomiting, or fever. There was no history of weight loss, cough, jaundice, pruritus, or any clinical feature of cholangitis. Laboratory studies showed a white blood cell (WBC) 10.1∗10^3^/μl with 74% neutrophils, ALT 61 U/L, AST 63 U/L, ALP 176 U/L, and a serum total bilirubin 18.2 μmol/L. Tumor markers were within normal limits with the exception of an SCCA of 1.8ng/mL. Urinary routine and microscopy examination were normal. Blood cultures were sterile. HBsAg and HBeAb were positive.

**Figure 1:**
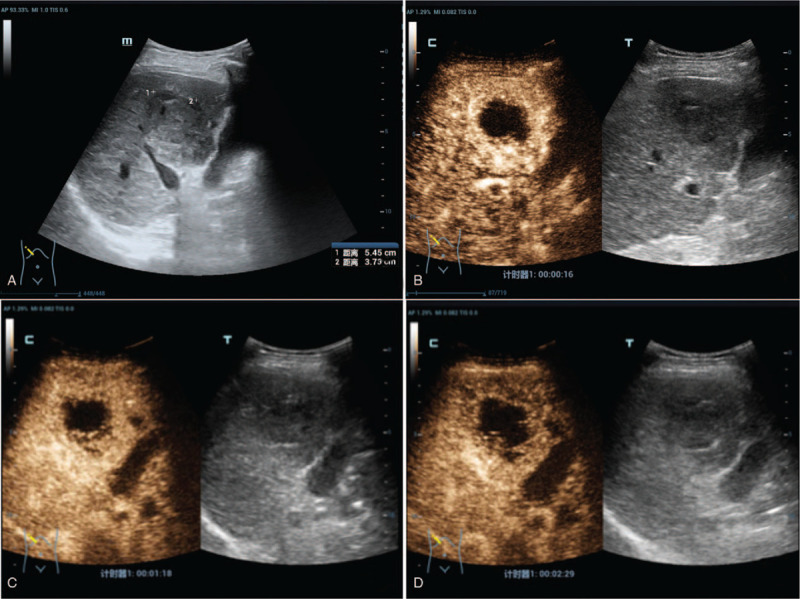
First contrast enhanced ultrasound. A, the hepatic V segment contained a hypoechoic lesion of size 5.1 cm ^∗^ 4.0 cm; B, arterial phase (16 seconds after SonoVue injection) showed an irregular, thick, circular hyper-enhancement with no enhancement in the inner lesion; C, portal venous phase (78 seconds) showed washout of the surrounding tissue around the lesion; D, venous phase (149 seconds) showed hypo-enhancement of the surrounding tissue around the lesion.

**Figure 2:**
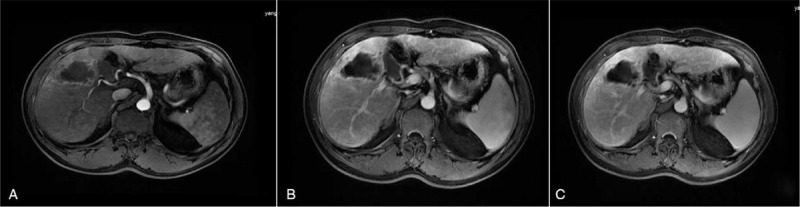
magnetic resonance imaging images. A, the hepatic V segment showed a rim-like enhancement of the irregular margin with no enhancement of the central cystic necrosis; B, the portal venous phase revealed decreased enhancement of the irregular margin; C, the venous phase showed substantial enhancement of the surrounding tissue.

Ultrasound (US) in our hospital revealed a hypoechoic lesion of 5.1  by 4.0 cm in the hepatic V segment with a thick, irregular and shaggy margin. There was no evidence of intrahepatic biliary radical dilatation and the common bile duct appeared normal. Contrast-enhanced ultrasound (CEUS) detected an irregular, thick circular hyper-enhancement with no enhancement in the inner lesion during the arterial phase. Washout of the surrounding tissue around the lesion in early portal phase was observed with showed hypo-enhancement in the venous phase (Fig. 1). The CEUS pattern revealed rapid wash in and wash out consistent with the diagnosis of a hepatic abscess. However, a diagnosis of atypical ICC was not excluded. Enhanced magnetic resonance imaging (MRI) showed a significant enhancement of the irregular margin with no enhancement of the central cystic necrosis. The portal venous phase revealed decreased enhancement consistent with a hepatic abscess, but not excluded atypical ICC (Fig. 2).

Therefore, the first diagnostic, US-guided biopsy was performed (2019.2.22) and revealed hepatic coagulative necrosis and pseudolobuli formation with lobular fibrous tissue hyperplasia and ductular proliferation. Lab values were as follows: WBC 12.3∗10^3^/μl with 79% neutrophils and a total serum bilirubin 23.2 μmol/L. The patient received Sulperazone 2 g twice a day in our hospital. In order to avoid a false negative result on the first biopsy, the second diagnostic biopsy was performed alongside a liver abscess drainage (2019.2.26). CEUS-guided biopsy from the enhanced wall of the lesion and drainage from the abscess cavity revealed a thick, purulent material. A culture of the abscess was sterile and cytology revealed abundant pus only. Pathology studies showed fibrous tissue proliferation with focal acute, and chronic inflammatory invasion with small ductular proliferation and a section of atypical tissue. Immunohistochemistry (IHC) expressed Vimentin with a population of fibroblasts. Based on these diagnostic findings, the patient was treated as a case of a hepatic abscess. After biopsy, the patient reported intermittent fever and was treated with Sulperazone for approximately 10 days. His temperature was normal with a WBC of 11.2∗103/μl after antibiotic and symptomatic therapy. He was discharged from the hospital (2019.3.1) and continued on oral moxifloxacin for about 20 days.

**Figure 3:**
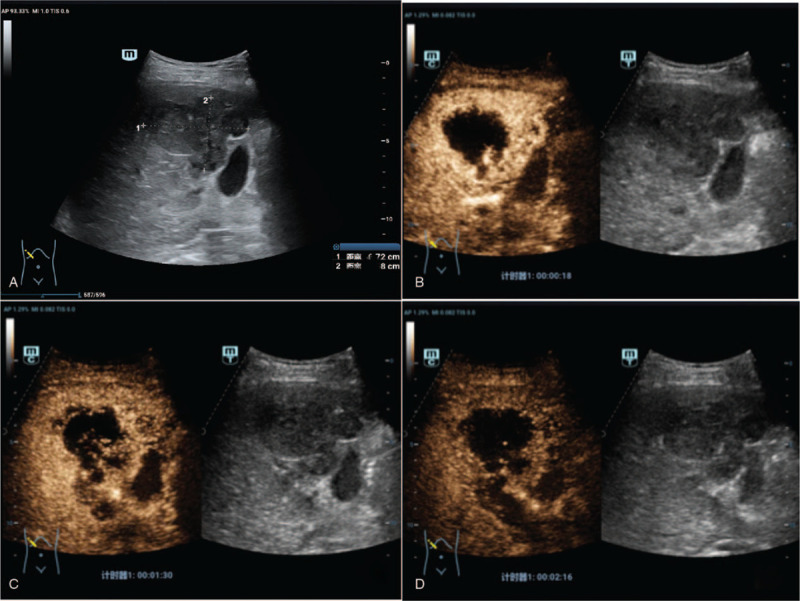
Third contrast enhanced ultrasound. A, the hepatic V segment showed a lesion of size 6.7 cm ^∗^ 4.7 cm, much larger than on initial imaging time; B, arterial phase (18 seconds after SonoVue injection) showed irregular, thick, circular hyper-enhancement with no enhancement area inner the lesion; C, portal venous phase (90 seconds) showed washout of the surrounding tissue around the lesion and enhancement of portal vein mass; D, venous phase (136 seconds) showed hypo-enhancement of the surrounding tissue around the lesion and portal vein mass.

**Figure 4:**
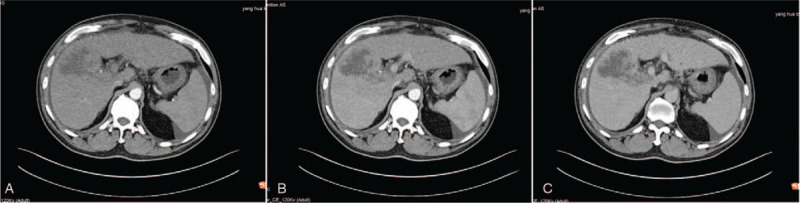
CT images. A, the hepatic V segment showed mixed density and irregular margin enhancement with no enhancement of the central cystic necrosis in arterial phase; B, the portal phase revealed decreased enhancement of the parenchymal tissue; C, the venous phase showed substantial enhancement of the margin.

**Figure 5:**
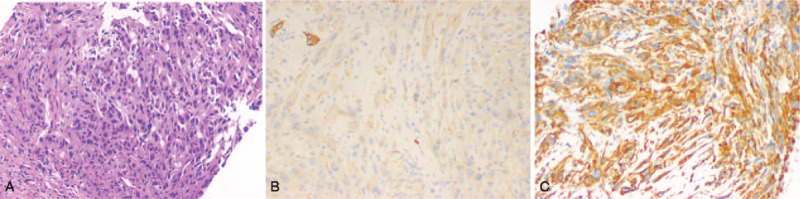
Pathological and immunohistochemical examination of a tumor sample. A, lesion tissue characterized by malignant spindle-shaped sarcomatoid hepatocellular carcinoma cells (HE×20); B, diffuse, weak positive signal for cytokeratin; C, diffuse strong positive signal for vimentin.

**Figure 6:**
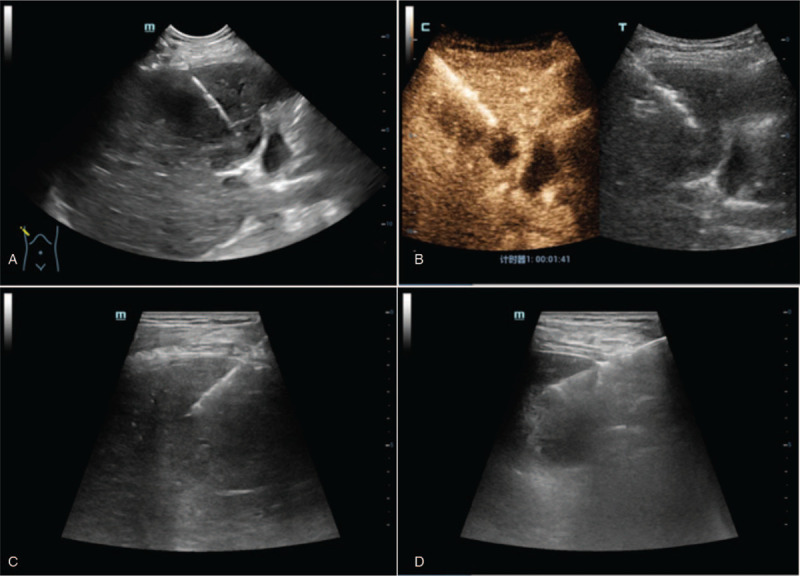
Ultrasound images of 3 performed biopsies. A, first time US-guided biopsy, the puncture needle was inserted alongside the margin of the lesion; B, second Contrast enhanced ultrasound-guided biopsy, the puncture needle was inserted at the enhancement wall of the lesion; C and D, third ultrasound-guided biopsy, the puncture was obtained from 2 different regions (hepatic VIII and V segments).

However, 20 days later, he presented to our hospital because of fevers up to 39.4 °C and severe abdominal pain (2019.3.20). Follow-up abdominal US and CEUS found a lesion of 6.7  by 4.7 cm in the hepatic V segment which was larger than on previous investigation. Enhancement was observed in the right portal vein without intra or extra-hepatic biliary extension (Fig. 3). Due to these findings, an etiology of ICC was considered and contrast-enhanced CT (Fig. 4) showed a solid, cystic mass of variable density, and no central enhancement due to necrosis. However, parenchymal tissue showed significant enhancement consistent with previous MRI. At this point, a third US-guided diagnostic biopsy was suggested (2019.3.28) and the following pathological diagnosis showed a poorly differentiated carcinoma with an IHC positive for cytokeratin (AE1/AE3) and vimentin (2019.4.17). With these findings, the diagnosis of SHC was finally confirmed (Figs. 5 and 6).

**Figure 7:**
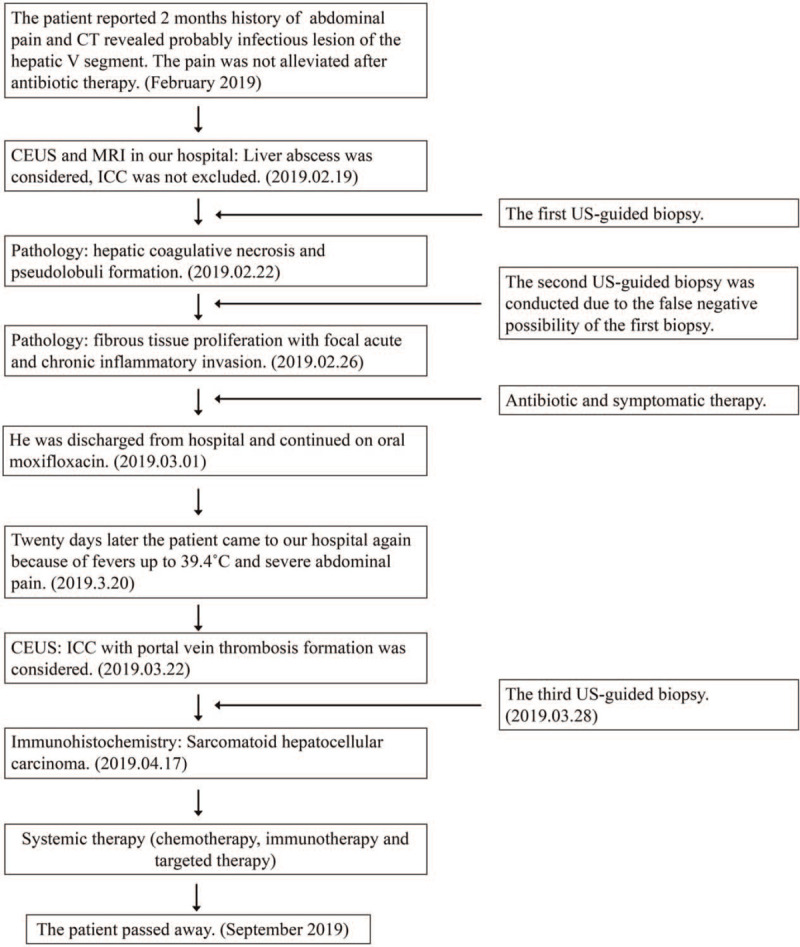
The flowchart of the treatment processes. CEUS = contrast enhanced ultrasound, CT = computed tomography, ICC = intrahepatic cholangiocarcinoma, MRI = magnetic resonance imaging.

The patient was not a candidate for surgical resection due to the presence of metastasis. Therefore, treatment with chemotherapy was initiated. The chemotherapy regimen consisting of Albumin paclitaxel (105 mg/m2, 200 mg i.v. for 30 minutes) and gemcitabine (850 mg/m2, 1600 mg i.v. for 30 minutes). After 4 courses of this regimen, tumor progression was found on enhanced MRI (2019.6.14). Therefore, the patient received immunotherapy and targeted therapy that consisting of PD-1 (130 mg/m2, 240 mg i.v. for 30 minutes) on day 1 and Anlotinib (10 mg po) on day 1 to day 14. The patient dead 3 months later due to tumor progression (September 2019). The flowchart of the patient's treatment process is as followed (Fig. 7).

## 3 Discussion

SHC is a relatively rare form of malignancy, characterized by features of epithelial and mesenchymal tumors. The fourth edition of WHO tumor classifications suggests that the sarcomatoid component represents a clonal evolution from a differentiated component from HCC or ICC.^[2,7]^ The etiology of SHC is not clear; some reports have suggested that HCC cases with sarcomatous changes after anticancer therapy such as transcatheter arterial chemoembolization, radiofrequency ablation, or percutaneous ethanol injection may cause the development of SHC, or accelerate the proliferation of sarcomatous cells.^[2,8]^ Some reports suggest that this uncommon subtype of liver cancer is also associated with hepatitis B and hepatitis C infection.^[3]^ Compared with HCC, the prognosis of SHC is much poorer due to frequent recurrence and metastasis. However, SHC is easily misdiagnosed as ICC and liver abscess, which delays the optimal treatment.

It is difficult to differentiate liver abscess from SHC not only in clinical practice but also on imaging examination. Clinically, shared features between the 2 pathologies include upper right quadrant abdominal pain, persistent fever with or without chills, rigor, nausea, vomiting, diarrhea, and weight loss. The nonspecific findings suggestive of a pyogenic liver abscess therefore delays the diagnosis of malignancy.^[2]^ Laboratory tests usually indicate an inflammatory reaction with increased WBC, erythrocyte sedimentation rate, and C-reactive protein in the liver abscess, which is also present in malignancy with infection and necrosis. Secondary liver abscess formation by SHC may be driven by tumor necrosis and subsequent infection and transformation to an abscess.^[4]^ As far as imaging examination is concerned, peripheral enhancement, and central necrosis are the shared features between SHC and hepatic abscess, complicating the diagnosis of these lesions. Central necrosis and hemorrhage of SHC as the sarcomatoid component consists of rapidly proliferating, poorly differentiated cells that outgrow their vascular supply.^[8]^ The tissue surrounding of the core of necrosis consists of inflammatory cytokines increasing arterial perfusion of the affected area. Therefore, SHC usually presents as a large mass with peripheral enhancement and central necrosis, variable enhancement of the solid portion with or without tumor capsule, and intra- and extra-hepatic metastasis.^[8]^ Alongside thrombosis of small portal and hepatic veins these mechanisms contribute to the observed increases in CEUS enhancement around the core of necrosis with a wash-out of the inflamed parenchyma.^[9]^ According to the findings presented herein, the most notable difference between a hepatic abscess and SHC is in the ring-enhancing part of the lesion, which is thicker in an abscess compared with SHC. Furthermore, hepatic abscesses usually demonstrate liquefactive necrosis in a honeycomb pattern without metastasis while SHC presents as diffuse liquefactive necrosis with metastasis or thrombosis of small portal and hepatic veins.

Additionally, it may be difficult to differentiate SHC from ICC by imaging without pathological confirmation. In most ICC cases, an irregular, rim-like hyper-enhancement is seen in the periphery of the lesion with a strip-like enhancement extending to the central portion in arterial phase. These findings are followed by a rapid washout in late arterial phase or early portal phase, and a marked washout in venous phase.^[10–12]^ The boundary of the non-enhanced area in ICC contains abundant fibrous tissue in the core of the tumor. The border between fibrous tissue and peripheral tumor cells is obscured due to the infiltrative tumor growth of cholangiocytes. However, the presence of intratumor vasculature is a defining characteristic of ICC.^[11]^ Despite the sensitivity of this marker, clinical diagnosis in practice is difficult. However, some ancillary findings may serve to differentiate SHC from ICC. In most cases of ICC, capsular retraction, bile duct dilatation distal to the lesion, and vascular encasement are very common. Meanwhile, intra and extra-hepatic metastasis are relatively common in SHC. Additionally an elevated CA19 to 9 with increases of ALP is more common in ICC than SHC, but these values are non-specific to either disease.^[4,10]^

In this case-study, a normal level of CA19 to 9 was found. Initial CEUS and enhanced MRI suggested a liver abscess followed by the presence of inflammatory and necrotic cells on guided biopsy. Due to the diagnosis of a hepatic abscess, surgery was not elected for this patient. Upon his second presentation to our hospital due to a fever of 39.4 °C with severe abdominal pain, CEUS suggested an ICC due to the increased mass and portal vein thrombosis. Nevertheless, only the third US-guided biopsy confirmed the diagnosis of SHC according to IHC results, which was positive for both an epithelial marker CK (AE1/AE3) and mesenchymal marker (Vimentin). The potential causes of initial misdiagnosis may be due to the position of the puncture and tumor progression. The pathology of the first biopsy showed necrotic tissue, which is consistent with the necrotic core of the tumor. The second biopsy suggested fibrous proliferation and focal acute inflammatory invasion, consistent with the proliferating rim of the tumor. Aspiration from the abscess cavity revealed thick, purulent material suggesting an inflammatory reaction such as a hepatic abscess. However, the third puncture was obtained from 2 different regions, and revealed the presence of malignant cells.

The prognosis of SHC is poor with surgical excision being the best option for effective treatment. Currently, the 3-year survival rate is as low as 18.2% after liver resection.^[13]^ Due to the presence of portal vein metastasis in this case, systemic treatment was the only viable option. However, had a definitive diagnosis been made earlier, surgical resection may have been possible.^[10]^ This case highlights the importance and difficulties in reaching an accurate and timely diagnosis of SHC.

## 4 Conclusion

In summary, this study reports a rare case of SHC and the resulting follow-up using CEUS, CT, and MRI. Eventually, SHC was confirmed through 3 diagnostic US-and CEUS-guided biopsies over a 3-month period. Due to the delay in diagnosis, the patient was not a candidate for surgical resection and showed dissemination of the lesion to the portal vein. Therefore, malignancy should always be considered when a hepatic abscess fails to resolve following the standard course of treatment. The specific patient population at risk would include an elderly patient presenting with persistent abdominal pain and intermittent fever with no response to multiple courses of antibiotics. Additionally, US-and CEUS-guided biopsies of multiple areas within the lesion are invaluable in making a timely and accurate diagnosis.

## Acknowledgments

We would like to thank Kanlun Xu for his contribution to the review of all the drafts of the manuscript.

## Author contributions

**Conceptualization:** Zheng Yang, Yanan Zhao.

**Data curation:** Zheng Yang, Kun Lv, Yanan Zhao.

**Formal analysis:** Kun Lv, Minqiang Pan.

**Investigation:** Zheng Yang, Chao Zhang.

**Methodology:** Yanan Zhao, Shumei Wei

**Project administration:** Yanan Zhao.

**Resources:** Yanan Zhao.

**Supervision:** Kun Lv.

**Validation:** Zheng Yang, Chao Zhang.

**Visualization:** Minqiang Pan, Chao Zhang, Shumei Wei.

**Writing – original draft:** Zheng Yang.

**Writing – review & editing:** Zheng Yang, Yanan Zhao.

## References

[1] Torbenson MS Morphologic subtypes of hepatocellular carcinoma. Gastroenterol Clin North Am 2017;46:365–91. 28506370 10.1016/j.gtc.2017.01.009

[2] Wang QB Cui BK Weng JM Clinicopathological characteristics and outcome of primary sarcomatoid carcinoma and carcinosarcoma of the liver. J Gastrointest Surg 2012;16:1715–26. 22767081 10.1007/s11605-012-1946-y

[3] Yu Y Zhong Y Wang J Sarcomatoid hepatocellular carcinoma (SHC): a case report. World J Surg Oncol 2017;15:219. 29233162 10.1186/s12957-017-1286-1 PMC5728015

[4] Shah V Arora A Tyagi P Intrahepatic cholangiocarcinoma masquerading as liver abscess. J Clin Exp Hepatol 2015;5:89–92. 25941437 10.1016/j.jceh.2014.12.006 PMC4415198

[5] Giuliani A Caporale A Demoro M Silent colon carcinoma presenting as a hepatic abscess. Tumori 2007;93:616–8. 18338500 10.1177/030089160709300618

[6] Lee SH Kim KA Lee JS A case of primary neuroendocrine carcinoma of liver presenting with liver abscess. Korean J Gastroenterol 2006;48:277–80. 17060722

[7] Miettinen M Fietcher CDM Kindblom LG Bosman FT Carneiro F Hruban RH Mesenchymal tumours of the liver. WHO Classification of Tumours of the Digestive System. Lyon: IARC Press; 2010 249.

[8] Koo HR Park MS Kim MJ Radiological and clinical features of sarcomatoid hepatocellular carcinoma in 11 cases. J Comput Assist Tomogr 2008;32:745–9. 18830104 10.1097/RCT.0b013e3181591ccd

[9] Kunze G Staritz M Köhler M Contrast-enhanced ultrasound in different stages of pyogenic liver abscess. Ultrasound Med Biol 2015;41:952–9. 25701525 10.1016/j.ultrasmedbio.2014.12.001

[10] Li C Li G Miao R Primary liver cancer presenting as pyogenic liver abscess: characteristics, diagnosis, and management. J Surg Oncol 2012;105:687–91. 21952992 10.1002/jso.22103

[11] Chen LD Ruan SM Liang JY Differentiation of intrahepatic cholangiocarcinoma from hepatocellular carcinoma in high-risk patients: a predictive model using contrast-enhanced ultrasound. World J Gastroenterol 2018;24:3786–98. 30197484 10.3748/wjg.v24.i33.3786 PMC6127655

[12] Bohle W Clemens PU Heubach T Contrast-enhanced ultrasound (CEUS) for differentiating between hepatocellular and cholangiocellular carcinoma. Ultraschall Med 2012;33:E191–5. 22194045 10.1055/s-0031-1282029

[13] Hwang S Lee SG Lee YJ Prognostic impact of sarcomatous change of hepatocellular carcinoma in patients undergoing liver resection and liver transplantation. J Gast rointest Surg 2008;12:718–24. 10.1007/s11605-007-0393-7 17999122

